# Study of the Effect of Anodic Oxidation on the Corrosion Properties of the Ti6Al4V Implant Produced from SLM

**DOI:** 10.3390/jfb14040191

**Published:** 2023-03-29

**Authors:** Ada Orłowska, Janusz Szewczenko, Wojciech Kajzer, Karolina Goldsztajn, Marcin Basiaga

**Affiliations:** Department of Biomaterials and Medical Devices Engineering, Faculty of Biomedical Engineering, Silesian University of Technology, 41-800 Zabrze, Poland

**Keywords:** scaffold, porous implant, Ti6Al4V, SLM, anodic oxidation, biocompatibility

## Abstract

Additive technologies allowed for the development of medicine and implantology, enabling the production of personalized and highly porous implants. Although implants of this type are used clinically, they are usually only heat treated. Surface modification using electrochemical methods can significantly improve the biocompatibility of biomaterials used for implants, including printed ones. The study examined the effect of anodizing oxidation on the biocompatibility of a porous implant made of Ti6Al4V by the SLM method. The study used a proprietary spinal implant intended for the treatment of discopathy in the c4–c5 section. As part of the work, the manufactured implant was assessed in terms of compliance with the requirements for implants (structure testing—metallography) and the accuracy of the pores produced (pore size and porosity). The samples were subjected to surface modification using anodic oxidation. The research was carried out for 6 weeks in in vitro conditions. Surface topographies and corrosion properties (corrosion potential, ion release) were compared for unmodified and anodically oxidized samples. The tests showed no effect of anodic oxidation on the surface topography and improved corrosion properties. Anodic oxidation stabilized the corrosion potential and limited the release of ions to the environment.

## 1. Introduction

Titanium and its alloys (including Ti6Al4V) are materials widely used in medicine. Due to their properties, i.e., favorable plastic and strength properties, high corrosion resistance and biocompatibility, electromagnetic properties, availability and processing ability, they are widely used [1,2]. In orthopedy, they are used to manufacture permanent (joint endoprostheses) and temporary (plates and stabilizers) implants, stents, screws, etc. [3]. Titanium alloys are also a material that can be surface modified, thus providing the products with even better properties. The modification methods include acid etching, anodic oxidation and plasma treatment, as well as coating with layers, including inorganic layers [4,5,6].

Thanks to the development of technology, new production methods, such as additive methods, are available. 3D printing, including the SLM method, is widely used in all branches of science and industry [7]. Elements produced with additive methods are increasingly entering operating theaters [8]. Additive methods enable the production of elements impossible to obtain with conventional methods [9,10]. Scaffolds and high porosity implants enable the creation of cell scaffolds that better replace the removed tissues and enable a more stable connection of the implant with the surrounding tissues [11,12,13]. Additive methods make it possible to produce elements with a strictly defined porosity and architecture [14,15,16,17]. The research has shown that the size and shape of the pores affect the ingrowth and maturation process of the bone tissue inside the implant. Obtaining mature bone tissue inside the implant provides an extremely stable, durable connection that prevents relocation of the implant [15,18,19,20]. In addition, this type of structure is characterized by more favorable mechanical properties and allows to avoid stiffening of the system and degradation of the bone tissue surrounding the implantation site [15,19,21,22]. Good mastery of the technique of producing implants with the use of additive methods also allows for the development of personalized medicine. 3D printing, supported by reverse engineering and CAD, allows for the production of implants ideally suited to the needs and anatomy of the patient [15,19,23,24].

Degradation of spinal structures is a common phenomenon, and the incidence of changes increases with age [25]. The treatment of spinal lesions depends on the stage of the lesion. In the case of significant degradation of the vertebrae or intervertebral discs, there may be pressure on the nerves, which results in chronic pain, numbness and sensory and locomotor problems. Surgical treatment consists in replacing the damaged tissue fragment with a fixed implant (arthrodesis) or a mobile implant (arthroplasty), which is to provide support to the structures and prevent compression [26]. Insertion of a fixed implant causes fusion of the vertebrae and a slight decrease in the mobility of the spine, but it is usually well tolerated. For a large group of patients, vertebral fusion provides a better therapeutic effect than treatment with a mobile implant while reducing potential complications [27,28,29,30]. Implants intended for spondylodesis are usually made of PEEK, less often of carbon fiber and titanium. Another solution is bone autografts taken from the iliac crest, but this procedure carries a higher risk and involves a wider operating field due to the need to collect material from the patient. One of the main risks when using implants of this type is obtaining an unstable or incorrect mechanical system at the implantation site. While solid implants made of PEEK are more similar in elasticity to bone tissue, studies have shown that the tissue surrounding the implant is very often characterized by high fibrosis, a thick layer of biofilm and an unstable implant-bone connection. This may lead to the relocation of the implant or the formation of pseudoarthrosis and the need for reoperation [13]. Replacing a solid implant with a scaffold, the porosity of which allows complete overgrowth of the implant with bone, gives hope for a better, more durable fixation. Particularly beneficial seem to be implants made of titanium alloy, which is characterized by high osteoconductivity. Highly porous titanium implants, despite the fact that the titanium alloys themselves have a higher Young’s modulus than bone, can be characterized by properties that perfectly imitate healthy bone tissue [15,18,21,22].

In clinical practice, there are numerous publications on the use of titanium implants produced in SLM technology [21,22,31]. However, these implants are not subjected to any additional surface modification, and their post-production processing is limited to cleaning, thermal treatment and sterilization. From the research of biomaterials, it is known that there are a number of techniques related to chemical, electrochemical and mechanical treatment that can ensure better biocompatibility, stability and corrosion resistance of metal implants. Modification of the surface of biomaterials is an important issue in biomedical engineering. The top layer in direct contact with the tissues affects the biocompatibility of the implant. The tissue response translates into the dynamics of the response at the cellular level, i.e., adhesion, migration, proliferation, differentiation leading to tissue maturation and bone formation [32,33]. Properly developed surface and roughness stimulate the differentiation of osteoblasts responsible for bone maturation and the formation of a stable implant-bone connection [34].

Despite all the advantages of SLM high-porosity implants, some caution is required. Although the properties and methods of modification of the Ti6Al4V alloy seem to be well tested, along with the change of the production method, it is necessary to verify. Surface modification, which has been successfully applied to items produced in a conventional manner [5,6,35,36], may not be optimal for the same material processed in the 3D printing process. Therefore, it is necessary to continue research and check whether the methods of modification of biomaterials, well researched by many scientists, still work well in the case of 3D printing [4]. The existing few works on the modification of implants printed from titanium alloys will not exhaust the subject, and the works on the clinical application of this type of implants are based on unmodified implants [21,22,31,37,38,39].

The aim of the work was to determine the effect of anodic oxidation on the corrosion properties of spinal implants made of Ti6Al4V produced by the SLM method. In particular, the influence of the environment imitating the tissue environment on the corrosion potential was determined and the number of ions released into the environment was examined. In addition, the microstructure of the implants, true porosity (micro-CT, helium pycnometry) and surface structure were assessed.

The obtained test results will allow to increase the corrosion resistance and, thus, the biocompatibility of the implants produced by the SLM method. Currently, the standard is the use of implants printed without the use of surface modification. These modifications for implants produced by conventional methods have a positive effect on the biocompatibility of the implanted elements.

## 2. Materials and Methods

### 2.1. Sample Preparation

**Figure 1:**
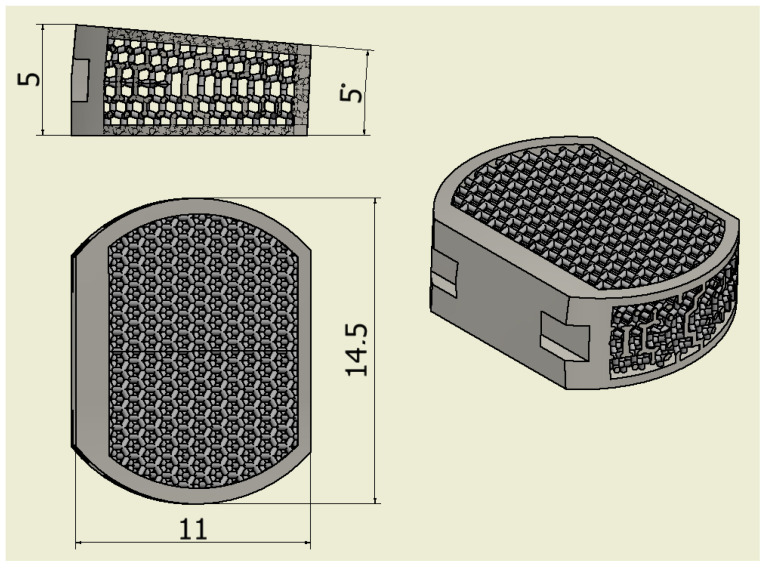
Model of the designed implant.

The samples were designed using INVENTOR 2020 (AutoDESK, San Rafael, CA, USA) (Figure 1). The shape of the implant enables it to replace the intervertebral disc in the C4-C5 segment. The sample was given an internal, open, highly porous structure based on a diamond mesh that allows for spherical inter-beam spaces. The pore size was determined to be 600 µm. The mesh and size of the pores were selected based on the literature analysis [14,15,16,17].

**Figure 2:**
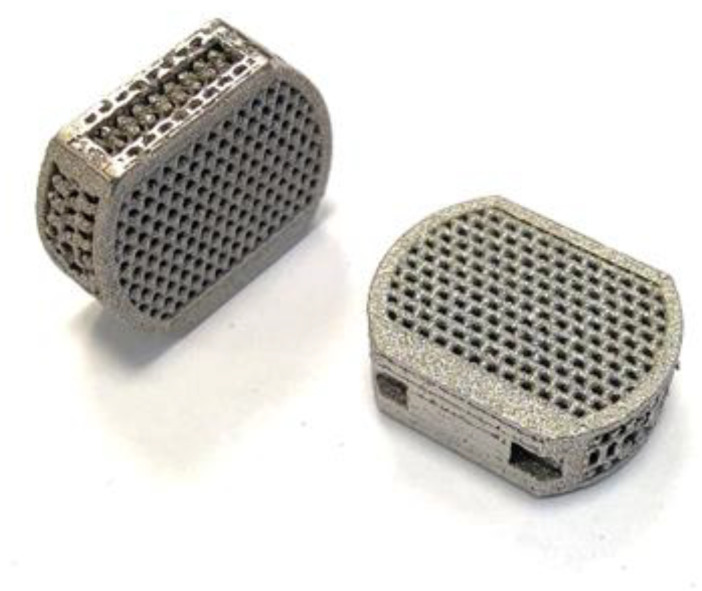
Implant produced by the SLM method.

The samples (Figure 2) were produced at ChM sp. z o.o. in Białystok on SLM-250 Metal 3D Printer (SLM Solutions Group AG, Lubeka, Niemcy) with the use of Ti6Al4V powder (TLS Technik GmbH, Bitterfeld-Wolfen, Germany). Finished samples were annealed at 920 °C for 4 h and then cleaned in isopropanol with ultrasound. After receiving the samples, their mechanical strength was verified with the use of a static compression test in order to demonstrate the resistance to physiological mechanical loads occurring in the body area dedicated to the implant [39].

### 2.2. Structure Evaluations

Metallurgical microsections were prepared for the produced samples. A resin (PolyFast, Struers, Cleveland, OH, USA) was used to enable SEM imaging. The samples were embedded in such way that it was possible to image longitudinal and transverse sections. Abrasive papers with gradations of 120, 320, 600, 800, and 1200 were used for grinding. The samples were polished with OP-S SUSPENSION (Struers, Cleveland, OH, USA) and etched in 8% HF acid. The photos of the structures were taken using a scanning microscope (TESCAN VEGA, SE detector, Brno, Czech Republic). The structure was assessed in accordance with ISO 20160.

### 2.3. Surface Modification

The produced samples were divided into implants in the initial state and modified state. The test group was subjected to anodic oxidation using the Titan Color solution (phosphoric acid + sulfuric acid). The samples were oxidized at the voltage U = 97 V for 2 min at room temperature.

### 2.4. Surface and Porous Structure Characteristics

The dimensional compliance of the produced samples was checked to assess the correctness of the printout. Due to the extensive internal structure, the samples were cut with a precision cutter (SECOTOM 15, Struers, Cleveland, OH, USA) with diamond friction so that the central part of the implant could be assessed. A digital microscope (Zeiss SteREO Discovery.V8, Oberkochen, Germany) was used to measure the sample size, pore size and pore shape. The measured values were compared with the dimensions of the model. In addition, for a more accurate assessment of the surface morphology, SEM images (TESCAN VEGA, Brno, Czech Republic) were taken.

### 2.5. Real Porosity Assessment

Porous structure and real porosity were assessed by micro-CT (CT-Compact, Casp system, Jaworzno, Poland) and helium pycnometry (1305 Micromeritics, Norcross, GA, USA). Based on the tomography scans, the actual shape and size of the pores, especially in the central part of the implant, were measured. The expected volume and the apparent volume necessary to determine the actual porosity were defined in the INVENTOR on the basis of the geometric model that was used during the sample production. The determined real volume was compared with the value of the expected volume.

### 2.6. Incubation of Samples

In order to evaluate the influence of environmental factors on implant degradation, the samples were incubated in PBS solution (0.14 M NaCl, 2.7 mM KCl, 0.01 M PO_4_^3−^, pH 7.4). The initial and anodized samples were placed in sealed sterile containers filled with 0.1 dm^3^ of incubation solution and placed in an incubator at 37 °C for 14, 28 and 42 days.

### 2.7. Corrosion Potential Assesment

The assessment of the corrosion potential was performed with the use of a potentiostat VoltaLab PGP 201 (Radiometer Analytical SAS, Villeurbanne Cedex, France) with VoltaMaster4 software (Radiometer Analytical SAS, Villeurbanne Cedex, France). A platinum electrode (auxiliary electrode) and a AgCl electrode (reference electrode) were used for the study. The study was carried out in PBS at 37 °C. Measurement of polarization curves was carried out for 2 h for each of the samples.

### 2.8. Ion Release Assessment

The mass density of ions released into the solution was assessed using plasma atomic emission spectroscopy (spectrometer ICP-AES JY 2000, HORIBA JOBIN YVON GMBH, Bensheim, Niemcy). The tests were carried out on filtrates with samples in their original state and anodized for 14, 28 and 42 days. The studies assessed the amount of Ti, V, and Al ions in the solution. The results were converted to the density of ions released from the sample surface. Standard curves prepared with the use of Merck standard materials were used to carry out the measurements.

## 3. Results

### 3.1. Structure Evaluations

**Figure 3:**
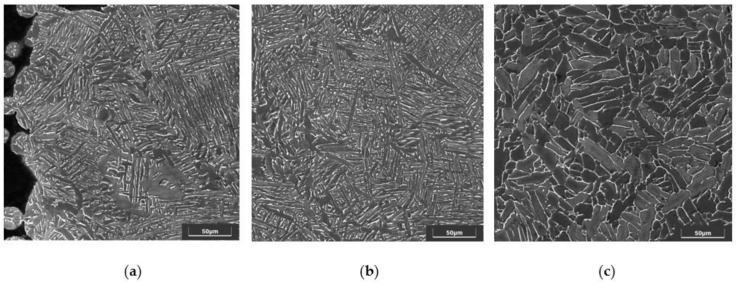
Microstructure of the material: (**a**)—for the solid part of the frame in the transverse plane; (**b**)—for the beams from the porous part in the transverse plane; (**c**)—for the solid part in the longitudinal plane; SEM.

The microscopic observations (Figure 3) show the microstructure of the material in the transverse plane for the solid part of the frame (a) and for the beams from the porous part (b) and in the longitudinal plane (consistent with the direction of building the element during production) for the solid part (c). The obtained images confirm the α+β structure of the tested material. SEM images were compared with standards from the norm. The structure in the longitudinal and transverse planes showed a slightly different character and corresponded, respectively, to the A3 and A4 standards from the ISO 20160 and ISO 52908:2022 standard.

### 3.2. Surface and Porous Structure Characteristics

**Figure 4:**
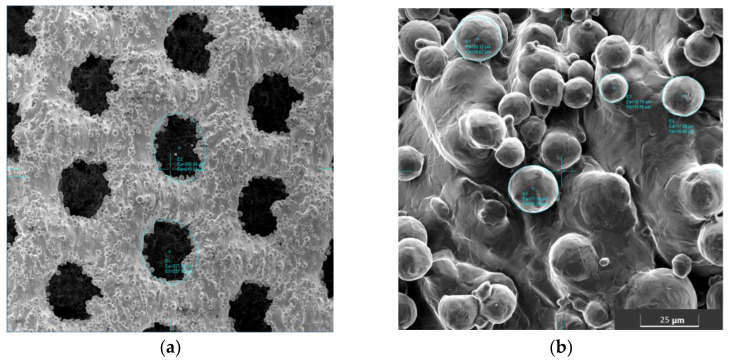
(**a**) Sample surface with measured pore size; (**b**) sample surface with a marked amount of powder deposited on the surface, SEM.

The macroscopic observations showed that in the produced samples, it was possible to obtain an open porous structure. The microscopic evaluation showed that the pores produced are spherical and slightly flattened, and their size is 450–600 µm (Figure 4).

The size of the measured pores was reproducible for all samples, both for the outer layers of the sample and in its central part. Although the pores produced differ in size from the intended value (600 µm), their size is within the range of values favoring the growth of bone tissue through the porous implant.

**Figure 5:**
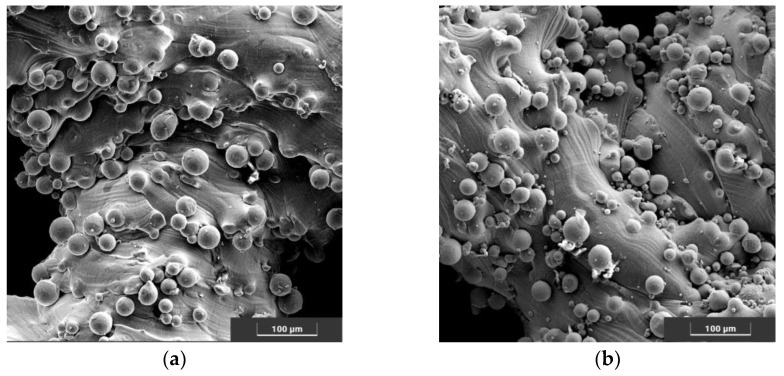

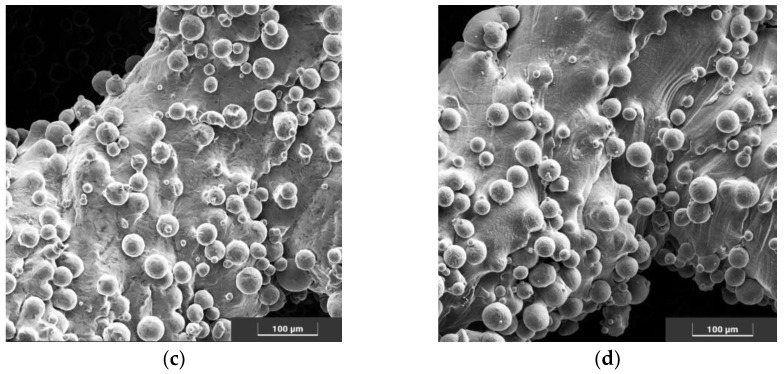
Surfaces of the implants produced before incubation in the (**a**) initial state, (**b**) anodically oxidized and after incubation in PBS in the (**c**) initial state, (**d**) anodically oxidized, SEM.

Comparing the SEM images of as-is and anodically oxidized samples, the modification had no effect on the amount of powder deposited on the surface of the sample (Figure 5).

Observations on the cut samples showed no significant differences between the top and inner layers of the scaffold. At the intersection site, no material discontinuity was observed in the solid beam structures, and no closed pores were present.

### 3.3. Real Porosity Assessment

**Figure 6:**
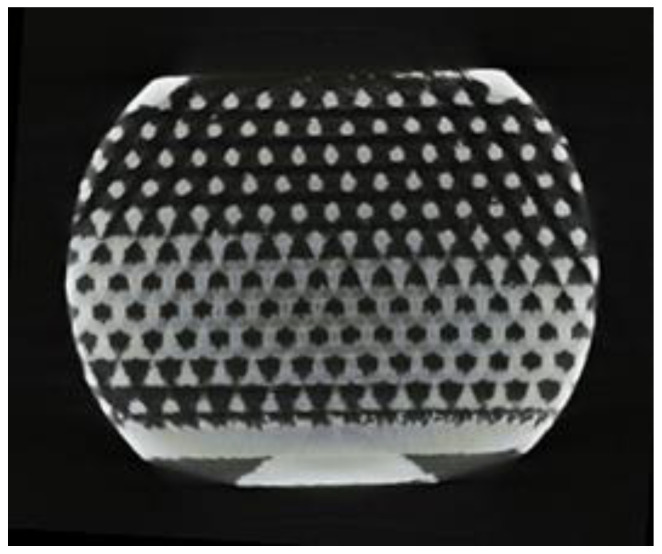
Transverse scan of the sample taken during micro-CT from the central part of the implant.

The micro-CT images (Figure 6) showed a uniform pore geometry throughout the sample volume. The scans obtained were confirmed by microscopic observations. The pores produced throughout the sample are open and spherical. The examination did not show the presence of closed pores or narrow structures impossible to colonize by bone tissue. Based on the geometric models in the INVENTOR program, the porosity of the samples was estimated at 65%. Helium pycnometry showed that the samples obtained had 49.0(10)% porosity. The density of the material determined on the basis of the actual volume and weight of the samples was convergent with the density characteristic for Ti6Al4V.

### 3.4. Corrosion Potential Assessment

**Figure 7:**
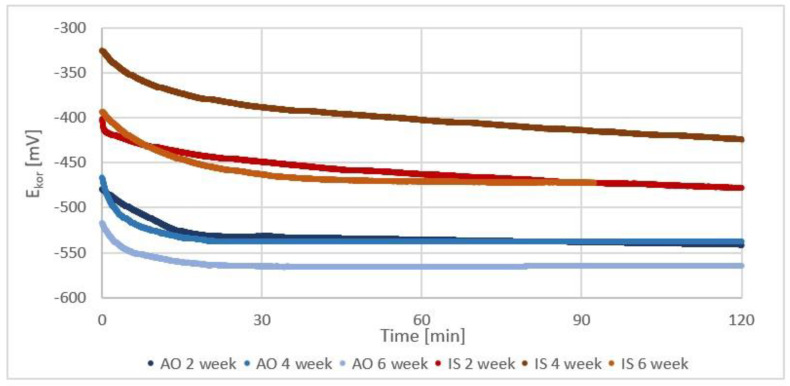
Sample corrosion potential curves for initial state (red) and anodized samples (blue).

The determined polarization curves showed that anodic oxidation significantly influenced the corrosion properties of the implants (Figure 7, Table 1). The corrosion potential of the samples in the initial state was close to zero. The corrosion potential of anodized samples was positive.

The tests carried out after 14, 28, and 42 days of incubation in PBS solution showed that the electric potential, both among the modified samples and in the initial state, decreased and became negative. It is worth noting that the determined corrosion potentials in the following weeks for anodized samples have a constant value. For samples in the initial state, the highest decrease in the value of the corrosion potential was determined for samples incubated for 14 days. In the following weeks, a gradual return to pre-incubation values was observed.

### 3.5. Ion Release Assessment

**Figure 8:**
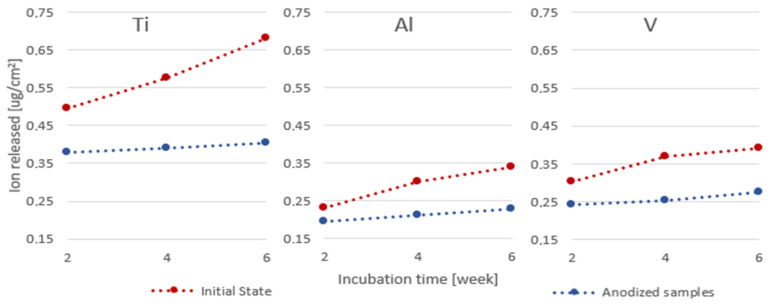
Graph of the ion density released into the incubation solution over time for initial states and anodized.

The conducted research has shown that the anodic oxidation of Ti6Al4V scaffolds produced by the SLM method slows down the process of penetration of metal ions into the environment (Figure 8). In all the tested samples, the level of the determined ions was higher for the native samples. Considering that the tested Ti, V and Al ions were not present in the incubation solution before the sample was placed in it, the most significant diffusion of ions into the environment took place in the initial phase of incubation. The gains between weeks 2 and 4 and 4 and 6 were the same for both periods and were much lower for all the ions tested.

## 4. Discussion

Comparing the observed structures to the micrographs of the ISO 20160 standard, it was confirmed that the processed material is suitable for medical applications. The disclosed metallographic structure in the transverse and frontal planes is not the same, which can be considered characteristic of elements produced by the SLM technology [40]. Nevertheless, individual microsections were homogeneous and characterized by a fine-grained α + β structure.

The assessment of the pore surface and structure showed a decrease in the actual pore size in relation to the designed pore size. This discrepancy is a direct result of the production technique [13,14,15,38,41]. Importantly, the obtained pore size (450–600 μ) was within the range of pores which, in the studies by other teams, ensured optimal bone tissue growth. Pores < 400 μm do not provide enough space to obtain a well-vascularized and thus nourished tissue. On the other hand, the use of larger pores > 900 μm delays the maturation of bone tissue and a weaker structure of the extracellular matrix [16,38,41].

The observed spheres deposited on the surface, most likely originating from insufficiently melted powder used to produce this type of implant, were also observed in other studies [15,20,39]. Smoothing the surface is possible by using a higher annealing temperature (>1300 °C), which could be observed, among others, in the studies of N. Taniguchi [16]. However, the use of such high temperatures can significantly affect the structure and, thus, the properties of Ti6Al4V. In the future, in-depth studies are planned on the effect of the annealing temperature of scaffolds produced from Ti6Al4V on the structure of the material and the properties of this type of implant. The observed structure of the surface carries a certain risk related to the possibility of grain detachment and its relocation in the body. On the other hand, obtaining an unstructured, highly developed surface may well stimulate osseointegration, constituting a favorable substrate for the migrating cells and the intercellular matrix they create. Studies conducted on similar scaffolds have shown that the proposed implants allow for a very stable implant-bone connection, which prevents licking and relocation of the implant [14,16,42]

Due to the reduced size of the actual pores, the actual porosity of the implant was also reduced. The research conducted by G. Li has shown that while the pore size has a significant impact on the way of the growth and maturation of bone tissue, the very ratio of the solid material to the pore volume does not affect the overgrowth of the implant [14].

Anodic oxidation caused the formation of a layer of TiO_2_ on the surface of the implant, which affected the surface properties of the implant. Due to the high stability of the TiO_2_ passive layer, this process is very beneficial because it increases the bio-compatibility of the material. The modified implants immediately after anodic oxidation showed a positive corrosion potential, which may indicate an improved corrosion resistance of the material [35,36]. However, the assessment of the corrosion potential in terms of the beneficial effect on the implant overgrowth with bone is not unambiguous. Bacakov’s research shows that the positive corrosion potential of the sample surface promotes the aggregation of osteoblasts [43]. Nevertheless, Anaselme research shows that it is beneficial to obtain a negative corrosion potential on the surface due to the need to promote protein aggregation on the surface of the material. This is beneficial not only in the initial phase of implantation (proteins aggregate on the surface earlier than cells) but also in the later phase, where in order to obtain a permanent implant-bone connection, not only is proper cell profiling on the implant surface necessary, but also tissue maturation, which is associated with the formation of the extracellular matrix composed mainly of proteins [42]. Comparing the corrosion potential of individual groups of samples after incubation in a liquid imitating the tissue environment, it can be seen that all groups showed a negative corrosion potential. Samples subjected to anodic oxidation showed higher stability, which should potentially provide better conditions for bone tissue overgrowth.

## 5. Conclusions

On the basis of the tests carried out, the designed implant was positively evaluated. Studies have shown that the implant is characterized by features that should translate into good osseointegration and stimulation of bone tissue to overgrow into the implant.

The proposed surface modification method using anodic oxidation had a positive effect on the properties of the Ti6Al4V implant produced by the SLM method. Oxidized implants were characterized by a more stable corrosion potential and limited penetration of metal ions into the external environment.

Further research is being carried out to optimize the methods of modifying implants made of titanium alloys produced by additive methods.

## Figures and Tables

**Table 1:** The average value of the corrosion potential E_cor_[mV] for individual groups of samples.

Time of Exposure, Weeks	0	2	4	6
Initial state [mV]	−11 (19)	−468 (11)	−430.0 (33)	−415.0 (20)
Anodic oxidated [mV]	403 (29)	−551.0 (60)	−547 (18)	−568 (12)

## Data Availability

The data presented in this study are available at the request of the appropriate author. The research results are presented in the article, and the data obtained during the tests are private.
